# Geomagnetic Navigation of Autonomous Underwater Vehicle Based on Multi-objective Evolutionary Algorithm

**DOI:** 10.3389/fnbot.2017.00034

**Published:** 2017-07-12

**Authors:** Hong Li, Mingyong Liu, Feihu Zhang

**Affiliations:** School of Marine Science and Technology, Northwestern Polytechnical University Xi'an, China

**Keywords:** geomagnetic navigation, bio-inspired navigation, geomagnetic anomaly, local optimal, evolutionary algorithm

## Abstract

This paper presents a multi-objective evolutionary algorithm of bio-inspired geomagnetic navigation for Autonomous Underwater Vehicle (AUV). Inspired by the biological navigation behavior, the solution was proposed without using *a priori* information, simply by magnetotaxis searching. However, the existence of the geomagnetic anomalies has significant influence on the geomagnetic navigation system, which often disrupts the distribution of the geomagnetic field. An extreme value region may easily appear in abnormal regions, which makes AUV lost in the navigation phase. This paper proposes an improved bio-inspired algorithm with behavior constraints, for sake of making AUV escape from the abnormal region. First, the navigation problem is considered as the optimization problem. Second, the environmental monitoring operator is introduced, to determine whether the algorithm falls into the geomagnetic anomaly region. Then, the behavior constraint operator is employed to get out of the abnormal region. Finally, the termination condition is triggered. Compared to the state-of- the-art, the proposed approach effectively overcomes the disturbance of the geomagnetic abnormal. The simulation result demonstrates the reliability and feasibility of the proposed approach in complex environments.

## Introduction

Autonomous Underwater Vehicle (AUV) has been widely used for both civilian and military applications, such as laying pipelines, ocean data collection, underwater equipment maintenance, and laying mines (Wadhams, 2012; Wynn et al., 2014; Shi et al., 2017). Most navigation systems rely on dead-reckoning that is by using inertial navigation system and velocity information from the Doppler sonar. However, the cumulative error becomes difficult to handle during the navigation, in contrast to the GPS and the acoustic transponder networks (Caiti et al., 2014).

Various techniques have been developed to eliminate the dead-reckoning errors (Hao et al., 2008; Yi et al., 2008; Shen et al., 2017). The geomagnetic navigation, which is originated from animal behaviors due to the earth's magnetic fields, has been widely used (Gould, 1984; Lohmann, 2010). It overcomes the following drawbacks: the error accumulation from the inertial navigation system, and the rapid attenuation signal from the satellite navigation system (Goldenberg, 2006). Thus, the geomagnetic field plays a key role in navigation, as it provides the position information in large scale environments (Teixeira and Pascoal, 2008). Typically, each point on the near-earth space has an unique magnetic field vector with respect to the corresponding coordinate, while the geomagnetic navigation is employed to provide a reliable navigation reference (Fu-qing, 2006; Zhou et al., 2008).

Based on the characteristics of the geomagnetism, numbers of methods have been developed (Caifa et al., 2011). The principle idea is employed based on the matching algorithms, such as MSD (Mean Square Difference), MAD (Mean Absolute Difference), ICCP (Iterative Closest Contour Point Algorithm) (Jia et al., 2012; Xie et al., 2013; Chong et al., 2014). However, traditional matching algorithms strongly depend on a priori geomagnetic map, which is quite challenging to acquire in practice.

To address this issue, the bio-inspired geomagnetic navigation method was proposed from animal behaviors (Liu et al., 2013). The most informative experimental paradigms have verified that the animals (like sea turtles and pigeons) can geomagnetic navigation to reach their goal locations (Paolo et al., 2003). Schulten concluded that animals can navigate by relying on geomagnetic sensitivity (Schulten, 1982). Winklhofer proposed the magneto-reception mechanisms to explain animals' navigation behavior (Winklhofer, 2009). Mole rats and salmons are also sensitive to the earth's magnetic field (Kimchi et al., 2004; Hays, 2013). In conclusion, the bio-inspired geomagnetic navigation provides a natural solution for navigation problem without a priori geomagnetic map.

**Figure 1:**
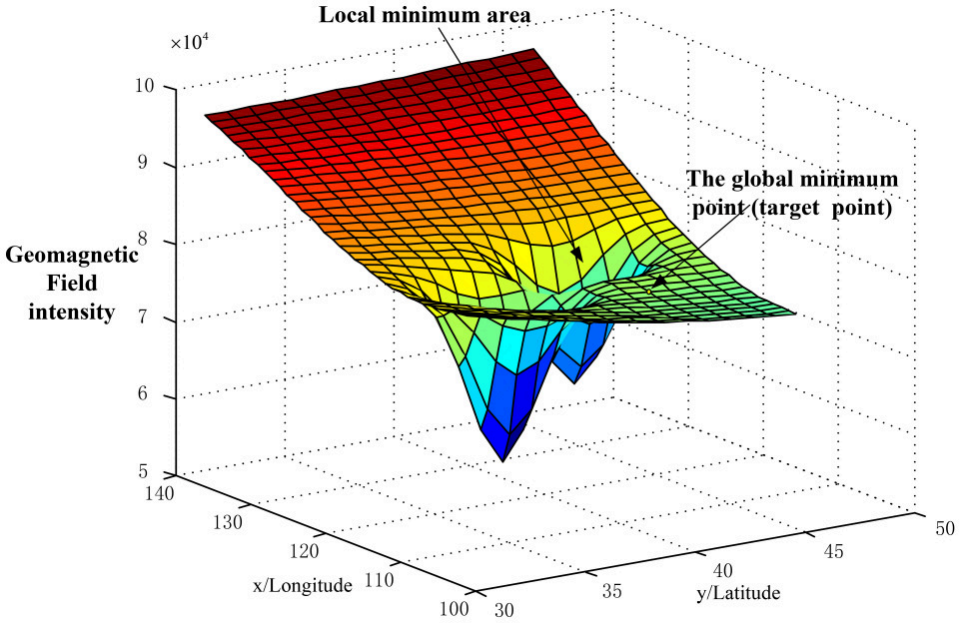
The geomagnetic anomaly scenario.

However, the geomagnetic anomaly often influences the bio-inspired geomagnetic navigation system which is caused by large iron ores. Boström showed that many geomagnetic blind spots were existed with respect to some migrating animals (Boström et al., 2012). Kiliowska pointed out the geomagnetic anomalies have interfered the whales' navigation system and caused them to beach (Klinowska, 1986). Lohmann pointed out the geomagnetic anomalies made the migrating turtles lost into Mexico (Lohmann et al., 2007). Dennis pointed out the pigeons were also easily lost when they were released in the geomagnetic anomaly area (Dennis et al., 2007). With respect to an AUV navigation, the geomagnetic anomalies disrupt the distribution of the geomagnetic field, which makes AUV lost in the navigation phase. In our previous works, the bio-inspired navigation algorithm was easily trapped in a local minimum point, caused by the geomagnetic anomalies (Liu et al., 2014). This happens where the distribution of the multiple geomagnetic was changed to the unsmooth and discontinuous regions (“concave” or “convex”). This caused the multi-objective search to converge to a local minimum easily (see Figure 1).

This paper presents a multi-objective evolutionary algorithm to address the local minimum problem by using the behavior constraint strategy. Main contributions are summarized as follows: First, it determines whether the algorithm is falling into the abnormal area. Second, it provides the strategy to jump out of the geomagnetic anomaly area. Once the algorithm is trapped in the local minimum, the behavior constraints are utilized to jump out the abnormal area.

The rest of this paper is organized as follows: Section Problem Formulation briefly introduces both the multi-objective search and the local minimum problems, respectively. Section Multi-Objective Evolutionary Algorithm with Geomagnetic Anomaly Presents the Multi-Objective Evolutionary Algorithm in Scenarios of Geomagnetic Anomaly. Section Numerical Simulations gives the main results and theoretical analysis. Finally, the conclusion is given in Section Conclusion.

## Problem formulation

In this paper, the motion of AUV on the horizontal plane is considered in the 2D Cartesian coordinate system. This simplification is justified due to the fact that the difference of the geomagnetic in vertical is often negligible. Therefore, the kinematic equations of the motion are introduced as follows:

(1) $$\{\begin{array}{l} x\left(k\right)=x\left(k-1\right)+v(k)\Delta T\text{ cos }\theta (k)\\ y\left(k\right)=y\left(k-1\right)+v(k)\Delta T\text{ sin }\theta (k) \end{array}$$

where Δ*T* is the sample period, *k* is the time instant, *v*(*k*) is the kinematic velocity, θ(*k*) is the heading of AUV in a time instant. Here, assuming AUV moves with a constant velocity, formula (1) can be written as follows:

(2) $$\{\begin{array}{l} x\left(k\right)=x\left(k-1\right)+L\text{ cos }\theta (k)\\ y\left(k\right)=y\left(k-1\right)+L\text{ sin }\theta (k) \end{array}$$

where *L* is the step size, *L* = *v*(*k*)Δ*T*.

### Mathematical description of the multi-objective search problem

The geomagnetic fields include multiple geomagnetic components (Caifa et al., 2011), which can be described as follows:

(3) $$B=\left\{B_{1},B_{2},\cdots ,B_{n}\right\}$$

where *B* is the set of the geomagnetic components vector, *B*_1_, *B*_2_, ⋯, *B*_*n*_ are defined as geomagnetic components, such as the north magnetic field *B*_*x*_, the east magnetic field *B*_*y*_, the downward magnetic field *B*_*z*_, the total intensity *B*_*F*_, the horizontal magnetic field *B*_*H*_, the declination angle *B*_*D*_, and the declination angle *B*_*I*_.

The bio-inspired navigation is thus the convergence process of the geomagnetic components from the current position to the target position (Liu et al., 2014). Therefore, the navigation process can be considered as the multi-objective searching problem as follow:

(4) $$\{\;\begin{array}{l} min\;F_{k}\left(f_{1}^{k}\left(B\right),f_{2}^{k}\left(B\right),\cdots ,f_{n}^{k}\left(B\right)\right)\\ s.t.\;f_{i}^{k}\left(B\right)={\left(B_{i}^{t}-B_{i}^{k}\right)}^{2},\;i\in n \end{array}$$

where $B_{i}^{t}$ is the geomagnetic component of the target position; $B_{i}^{k}$ is the geomagnetic component of the current position; $f_{i}^{k}$ is the difference of the *i*^*th*^ geomagnetic component between the target position and the current position; *F*_*k*_ is the objective function.

Considering the different magnitude of the geomagnetic components, the objective function is normalized as follows:

(5) $$F_{k}=\sum_{i=1}^{n}\frac{f_{i}^{k}\left(B\right)}{f_{i}^{0}\left(B\right)}=\sum_{i=1}^{n}\frac{{\left(B_{i}^{t}-B_{i}^{k}\right)}^{2}}{{\left(B_{i}^{t}-B_{i}^{0}\right)}^{2}}$$

The errors between the current position and the target position can be assumed as the geomagnetic trend, while the searching process is terminated when the error converges to ε. This is expressed as:

(6) $${\text{lim}}_{k\to \infty }\left\|F_{k}-F_{k-1}\right\|<\varepsilon$$

where ε is a fixed value.

The bio-inspired navigation is thus considered as a posteriori searching problem, in presence of unknown geomagnetic components. Once the geomagnetic error between the current position and the target position converges to zero, the navigation process is terminated.

### Geomagnetic searching in anomaly environment

The geomagnetic anomaly field has significant influences on the geomagnetic navigation system, where the related factors are analyzed according to the distribution characteristics of the geomagnetic field (Talwani, 1965). Usually the geomagnetic anomaly is given by:

(7) B′=B+ΔB

where *B* is the amplitude of the abnormal region.

Considering the geomagnetic anomaly for the bio-inspired algorithm, the difference of the *i*^*th*^ geomagnetic component is expressed as:

(8) $$f_{i}^{\prime_{k}}=\;{\left(B_{i}^{\prime_{t}}-B_{i}^{k}\right)}^{2}={\left(B_{i}^{t}+\Delta B-B_{i}^{k}\right)}^{2}$$

Combining formulas (7) and (8), the convergence condition is given by:

(9) $$\begin{array}{l} \left\|f_{i}^{\prime_{k}}-f_{i}^{\prime_{k-1}}\right\|=\left\|{\left(B_{i}^{t}+\Delta B^{k}-B_{i}^{k}\right)}^{2}-{\left(B_{i}^{t}+\Delta B^{k-1}-B_{i}^{k-1}\right)}^{2}\right\|\\ \;=\left\|{\left(B_{i}^{t}-B_{i}^{k}\right)}^{2}-{\left(B_{i}^{t}-B_{i}^{k-1}\right)}^{2}+2\left(B_{i}^{t}-B_{i}^{k}\right)\Delta B^{k}-\;2\left(B_{i}^{t}-\;B_{i}^{k-1}\;\right)\;\Delta B^{k-1}+{\left(\Delta B^{k}\right)}^{2}-{\left(\Delta B^{k-1}\right)}^{2}\right\|\\ \;\leq \left\|{\left(B_{i}^{t}-B_{i}^{k}\right)}^{2}-{\left(B_{i}^{t}-B_{i}^{k-1}\right)}^{2}\right\|+\left\|2\left(B_{i}^{t}-B_{i}^{k}\right)\Delta B^{k}-\;2\left(B_{i}^{t}-\;B_{i}^{k-1}\;\right)\Delta B^{k-1}+{\left(\Delta B^{k}\right)}^{2}-{\left(\Delta B^{k-1}\right)}^{2}\right\|\\ \;<\varepsilon +\left\|2\left(B_{i}^{t}-B_{i}^{k}\right)\Delta B^{k}-2\left(B_{i}^{t}-\;B_{i}^{k-1}\;\right)\Delta B^{k-1}\right\|+\left\|{\left(\Delta B^{k}\right)}^{2}-{\left(\Delta B^{k-1}\right)}^{2}\right\|\\ \;=\xi \end{array}$$

where ξ is bigger than ε if the geomagnetic anomalies exists. This result shows that the algorithm is easily trapped in the local minimization.

## Multi-objective evolutionary algorithm with geomagnetic anomaly

This section gives the general idea of the proposed algorithm in geomagnetic anomaly, the experiment will be given in next section.

### The search principle

A bio-inspired navigation method is investigated, which is based on a simple assumption that a homing animal only senses and compares the variation of the geomagnetic field to reach its home.

Evolutionary algorithm is often used to solve problems in highly complex spaces (Cliff et al., 1993; Droste et al., 2002; Vrugt and Robinson, 2007; Peng et al., 2016; Peng and Wu, 2017). Based on Darwin's evolutionary theory, ethologists have been concerned with the evolution of animals' behavior (Alerstam et al., 2003). Searching behavior can be described as active movement, in which animals attempt to find resources such as food, mates, nesting sites (Bell, 1992). Based on this, McFarland has proposed a number of mathematical analogs by using adaptive evolutionary behaviors (McFarland and Bösser, 1993).

Hence, by utilizing the evolution of animals behaviors, the evolutionary algorithm is employed for searching the geomagnetic space. In evolutionary algorithm, each possible solution is defined as the individual, whereas a set of individuals is defined as the population. The evolution method requires maintaining a population of various individuals, according to the operators such as selection, mutation, and so on. The repeated process of recombination, selection, and mutation leads the individuals to adapt to the environment, whereas the selection operator is implemented by a task-oriented evaluation function: the better the AUV performs its task, the more offspring it has.

However, the local anomalies from the geomagnetic field have significant influences on the proposed geomagnetic navigation system. The next section explains the solution to the anomaly problem.

### The search algorithm with geomagnetic anomaly

This section discusses how to deal with the local minimum problem within the geomagnetic anomaly areas. Our solution has two phases: the first phase is to determine whether the algorithm falls into the geomagnetic anomaly area, and the second is to determine whether the algorithm arrives at the target location.

The first phase refers to the environmental monitoring operator Ψ(*k*), which can be expressed as:

(10) $$\begin{array}{l} \text{Ψ}\left(k\right)=\frac{\left\|F_{k}^{\prime }-F_{k-1}^{\prime }\right\|}{\frac{1}{{\text{T}}_{1}}\sum_{i=1}^{{\text{T}}_{1}}\|F_{k}^{\prime }-F_{k-1}^{\prime }\|}=\frac{\left\|F_{k}^{\prime }-F_{k-1}^{\prime }\right\|}{E\left(\Delta F_{{\text{T}}_{1}}^{\prime }\right)} \end{array}$$

where T_1_ is the period of time, *E*(·) is described as mean values.

**Figure 2:**
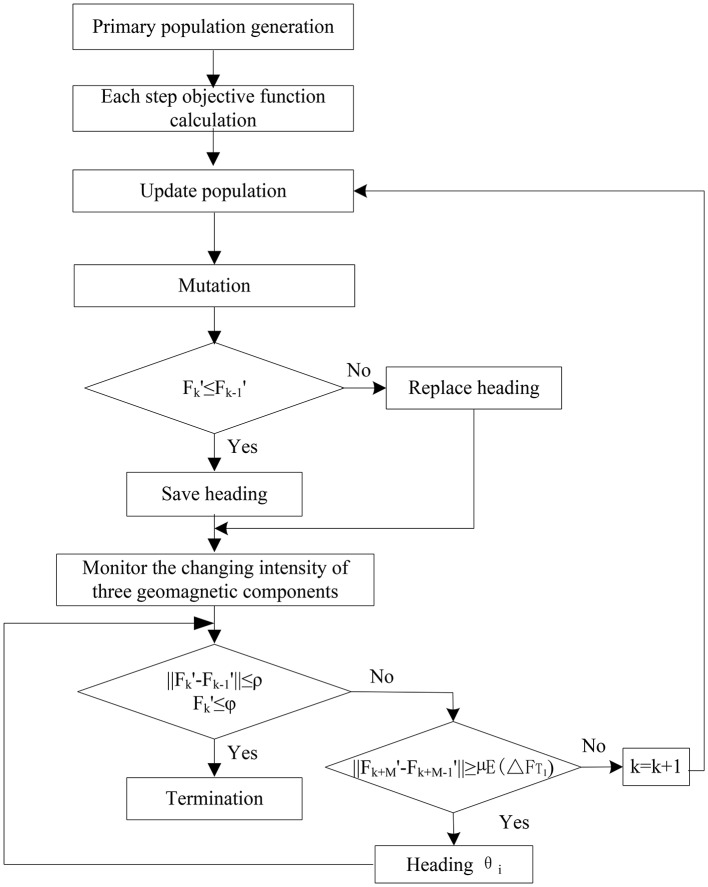
The workflow of the evolutionary search within geomagnetic anomaly.

The termination condition in second phase is determined as:

(11) $$\left\{ \begin{array}{l} \|F_{k}^{\prime }-F_{k-1}^{\prime }\|\;\leq \;\rho \\ \;F_{k}^{\prime }\leq \;\varphi \end{array}\right.$$

where ρ is a small value zero, φ is the scope of the desired target location. The steps for the multi-objective evolutionary algorithm which can address the challenge of the geomagnetic anomaly are given as follows:

**Step 1:** Population Initialization. Randomly generate *N* individuals in the population space *Q* (*Q* = {θ_*j*_|*j* = 1, 2, ⋯, *N* }), θ is the set of samples and given by:
  (12) $$\begin{array}{l} \theta =\left\{\theta_{1},\theta_{2,}\cdots {,\theta }_{m},\;m=\frac{2\pi }{\Delta \theta },\theta_{j}=\Delta \theta \times i,i\in \left[1,m\right]\;\right\} \end{array}$$
  where θ is the sampling interval.
**Step 2:** Individual Selection. Randomly select the sample θ_*j*_, and the probability of each individual is given by:
  (13) $$\begin{array}{l} p\left(\theta_{j}\right)=\;\frac{1}{N} \end{array}$$
  when the current number of iterations is *k*, the probability of the selected individual θ_*i*_ can be obtained as
  (14) $$p(\theta_{j}^{k})=\;\frac{\sum_{j=1}^{N}\delta_{j}(\theta_{j}=\theta_{i})}{N}$$
  where δ is a symbolic function.
**Step 3:** Population Updating Rules. The termination is calculated if the selected individual accomplishes a successful search. Thus the individual updating rules can be divided into two parts: for one that moves toward the target direction ($F_{k}^{\prime }\leq F_{k-1}^{\prime }$), the selected individual heading is reserved in the population. And for the other that moves away from the target direction ($F_{k}^{\prime }>F_{k-1}^{\prime }$), the selected individual heading is replaced.
  (15) $$\left\{ \begin{array}{l} \theta_{j}=\theta_{i},F_{k}^{\prime }\leq F_{k-1}^{\prime }\\ \theta_{j}^{k}\;=\;\Delta \theta \times i,\;F_{k}^{\prime }>F_{k-1}^{\prime } \end{array}\right.$$
**Step 4:** Environmental Monitoring Operator. The monitoring factor Ψ(*k*) is used to determine whether the algorithm falls into the geomagnetic anomaly area (Ψ(*k*) ≥ μ, μ is the threshold value).
**Step 5:** Motion Constraint Operator. Once the search algorithm is trapped into the local minimum area, the maximum probability θ_*i*_ is preserved. This can be expressed as:
  (16) $$\begin{array}{l} \theta_{i}^{k}=\;\theta_{max\left\{p\left(\theta_{i}\right)\right\}} \end{array}$$
  When formula (17) is satisfied, the algorithm jumps out of the local minimum area.
  (17) $$\begin{array}{l} \|F_{k+M}^{\prime }-F_{k+M-1}^{\prime }\|<\mu E\left(\Delta F_{T_{1}}^{\prime }\right) \end{array}$$
**Step 6:** Termination condition. The navigation is terminated if formula (11) is satisfied. The workflow of the proposed evolutionary algorithm is shown in Figure 2.

## Numerical simulations

To verify the effectiveness of the proposed multi-objective evolutionary algorithm, numerical simulations are performed.

### Simulation setup

The Word Magnetic Model (WMM2010) is used to provide the real time geomagnetic data (Maus et al., 2012). In simulation, a rectangular area is selected from north latitude 30° and east longitude 100° (N.30° and E.100°) to north latitude 45° and east longitude 135° (N.45° and E.135°). Considering the non-relevance of the geomagnetic components, only three geomagnetic components are used, which are the north magnetic field *B*_*x*_, the east magnetic field *B*_*y*_, and the total intensity *B*_*F*_, respectively.

**Figure 3:**
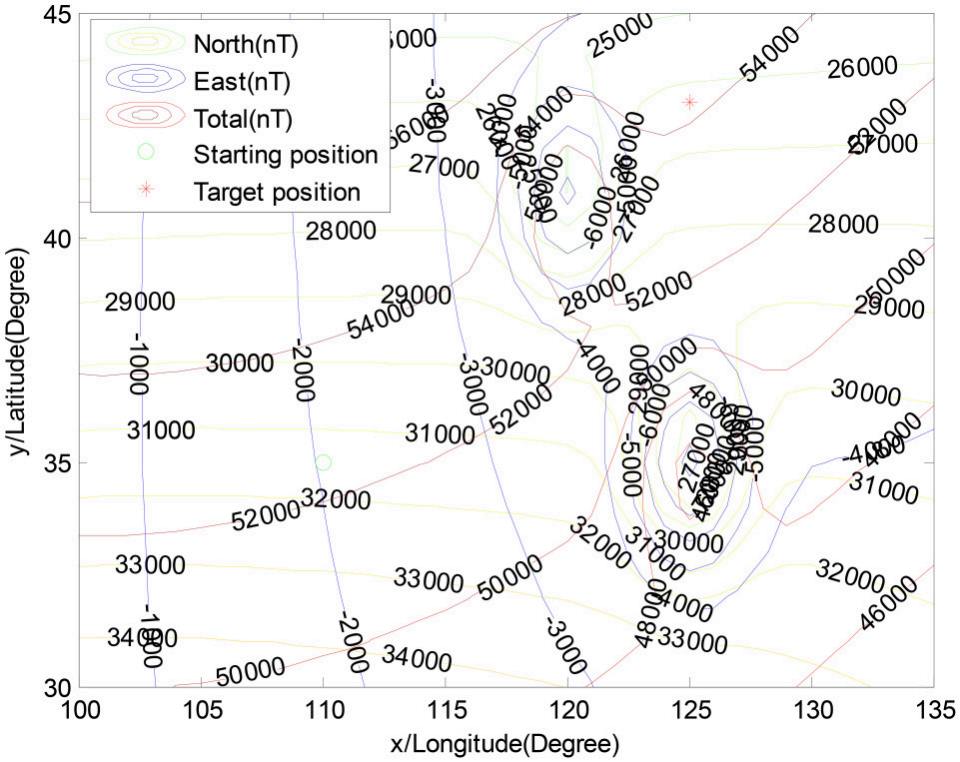
The simulation scenario within multi-modal function.

Meanwhile, it is assumed that the geomagnetic navigation would encounter a geomagnetic anomaly region. Here, the multi-mode function is utilized to construct the abnormal geomagnetic environment (see in Figure 3), where the intensity of the highest abnormal geomagnetic field value is −5,000*nT*.

In Figure 3, the starting position and the target position are depicted by using the three geomagnetic components at *B*^0^ = [31, 464*nT*, −2, 036*nT*, 52, 508*nT*] and *B*^*t*^ = [26, 290*nT*, −4, 229*nT*, 54, 254*nT*], respectively. The circle, “◦”, stands for the starting position, and the star, “^*^,” stands for the target position. The related parameters are shown in Table 1.

**Table 1:** Setting navigation parameters.

**No**	**Parameters**	**Size**
1	*L*	500 m
2	*N*	50
3	Δθ	30°
4	ρ	0.007
5	φ	0.01
6	μ	1.6
7	p_m_	0.02

### Simulation results

To evaluate the performance of the proposed approach, two cases are evaluated: one occurs without considering geomagnetic anomaly and the other one with geomagnetic anomaly.

**Figure 4:**
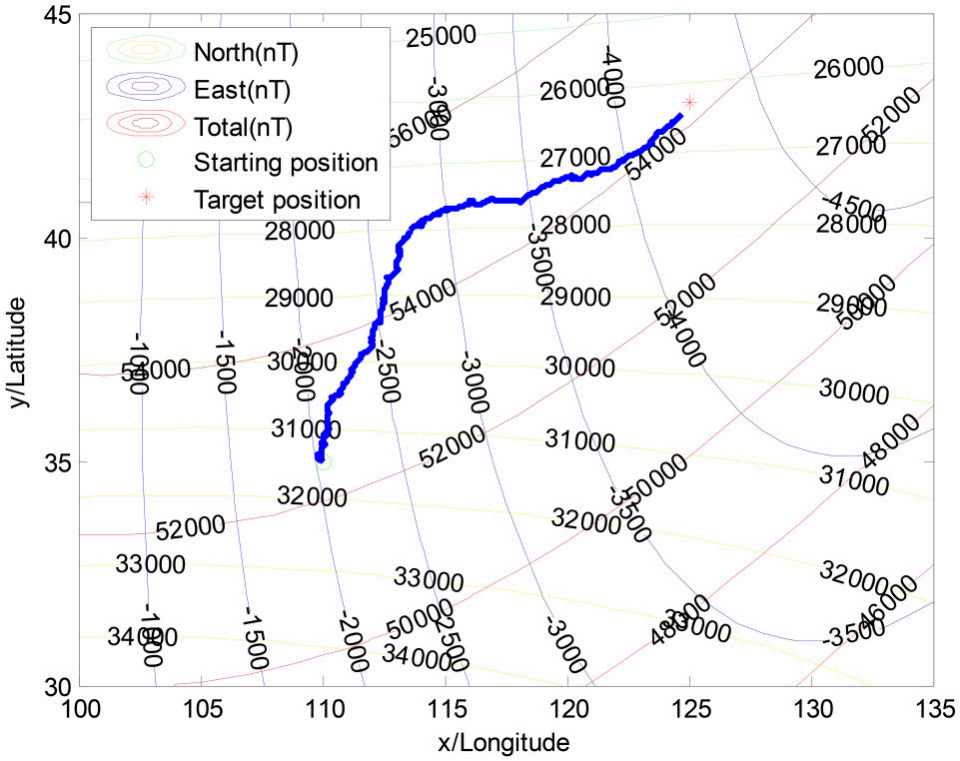
The navigation without the geomagnetic anomalies.

**Figure 5:**
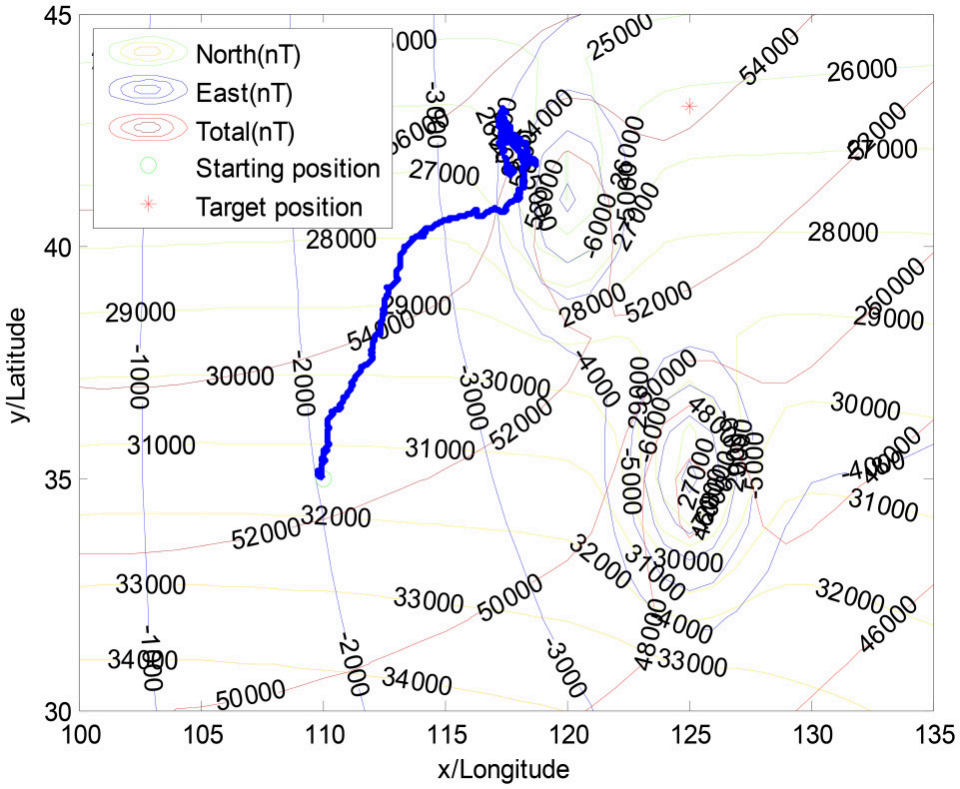
The navigation with the geomagnetic anomalies.

Figure 4 illustrates the navigation trajectory without the interference of geomagnetic anomalies, whereas Figure 5 illustrates the moving trajectory with the interference of geomagnetic anomalies. As shown in Figure 5, the previous algorithm is easily trapped into the local minimization in geomagnetic anomaly areas, in which individuals move toward different directions (Liu et al., 2014). It is observed that the navigation performance strongly depends on the geomagnetic anomalies.

It is concluded that the original searching algorithm is not capable for the anomalies of geomagnetic fields, in which AUV moves toward different directions and ultimately fails.

**Figure 6:**
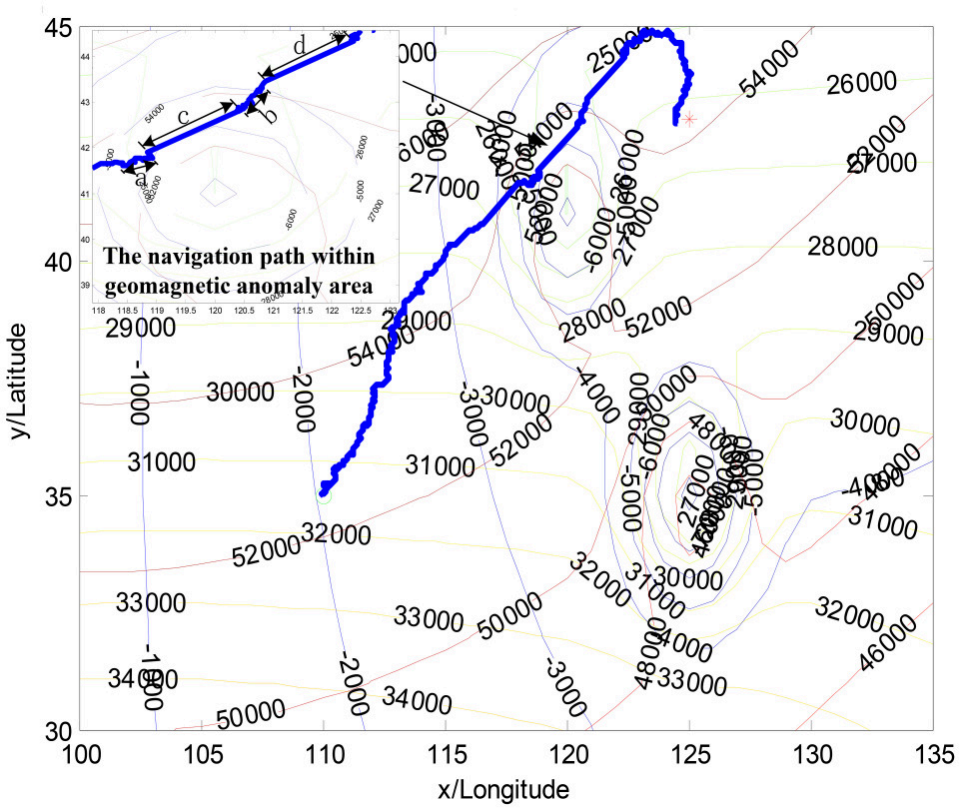
The navigation result in the geomagnetic anomalies with the proposed solution.

Figure 6 illustrates the proposed approach, where AUV successfully overcomes the influence of the geomagnetic anomalies. During the navigation phase, the individual directions are randomly changed due to the probability selected in the population. Zoomed figure on the left corner shows the two motion constraints, which are performed using the monitoring factor Ψ(*k*). Two time periods “c” and “d” are within the geomagnetic anomaly area, while “a” and “b” represent the variation anomalies of the geomagnetic components, respectively. By monitoring the changes of the geomagnetic components, the proposed approach effectively detects the abnormal regions. Then, the statistic characteristics of the convergence state is utilized to the behavior constraints, for the purpose of jumping out the abnormal region.

### Convergence performance of the search algorithm

**Figure 7:**
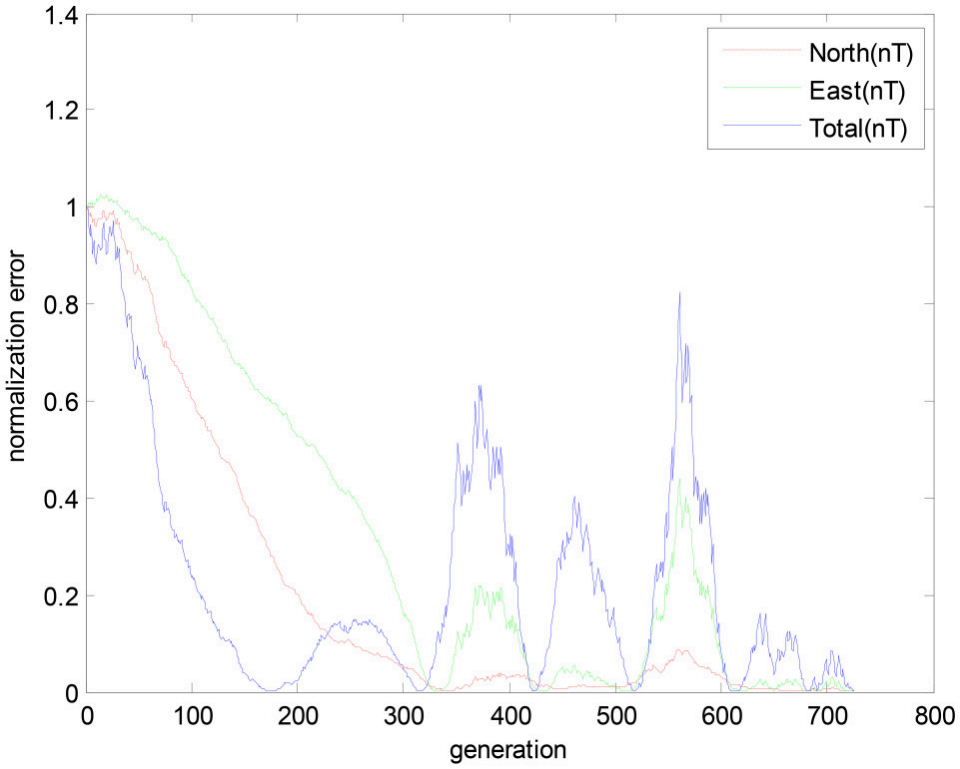
The convergence curves of geomagnetic components within the geomagnetic anomalies.

The convergence curves of three geomagnetic components are shown in Figure 7. It is observed that the convergence curves present violent shakings in three geomagnetic components.

**Figure 8:**
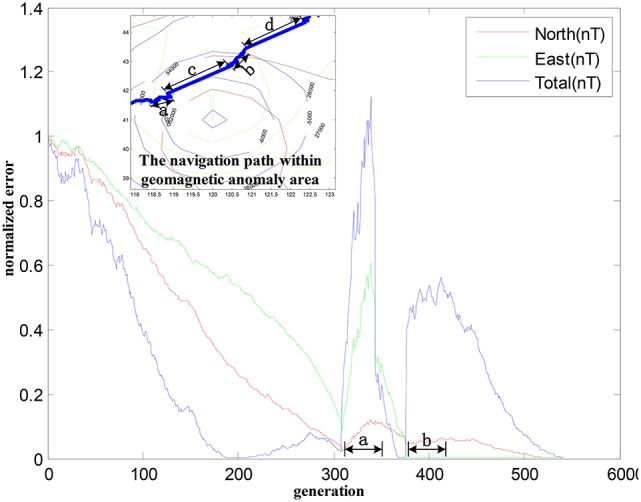
The geomagnetic components convergence within the geomagnetic anomalies.

To better evaluate the performance in presence of geomagnetic anomalies, the convergence properties of three geomagnetic components are provided. As depicted in Figure 8, there is a divergence trend for the three geomagnetic components in periods of “a” and “b.” The convergence properties of the geomagnetic components are improved after using behavior constraints, demonstrating that the normalized error are able to converge to a stable state. It is observed that the improved algorithm could converge the curves of the geomagnetic components to zero in finite steps.

**Figure 9:**
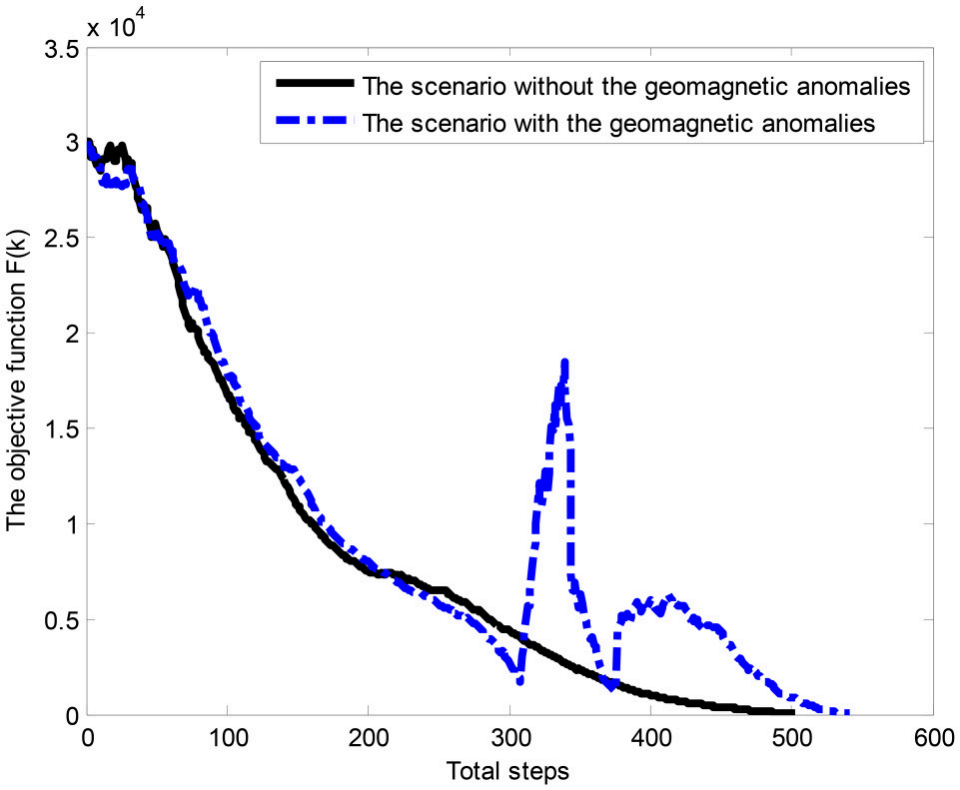
The convergence of the objective function.

To explore the advantages of the proposed algorithm, the traveling time employed by the AUV in both scenarios is also compared in Figure 9. It shows that the proposed approach could converge to a stable state in scenarios of geomagnetic anomalies, and the navigation process is terminated in 540 steps.

### Discussion

In this paper, the multi-objective evolutionary algorithm has been proposed to address the AUV navigation problem. As a result of geomagnetic anomaly, the distribution of the geomagnetic components is often disrupted over the searching space. Therefore, the environmental monitoring and the behavior constraints are used to tackle the local optimal problem. The time complexity of the proposed algorithm is O(*k*^3^) in the geomagnetic anomalies.

For the geomagnetic navigation, the optimal path between the starting position and the destination is quite challenging without a priori geomagnetic map. Also, the AUV navigation in oceans strongly influences by the geomagnetic anomaly effects. However, the proposed approach indicates that the searching strategy performs optimally for AUV navigation in the geomagnetic anomaly areas.

Thus, the multi-objective searching algorithm with the environmental monitoring and the behavior constraints is proposed to ensure the success and safety of AUV navigation.

## Conclusion

This paper presents a novel strategy for bio-inspired geomagnetic navigation in presence of geomagnetic anomalies. Inspired by the biological navigation, in our previous work we proposed an evolutionary schema which helps the AUV to reach the destination without geomagnetic map. However, it suffers the disturbance from geomagnetic anomalies and often converges to the local minimum point. To tackle the problem, this paper proposes an improved navigation model by introducing constraints strategy to make AUV escape from the abnormal regions. Simulation results show that the proposed model effectively overcomes the disturbance of the geomagnetic abnormal for AUV navigation.

## Author contributions

HL wrote the paper and performed the experiments. ML provided some ideas to improve and perfect the paper. FZ reviewed and edited the manuscript. All authors read and approved the manuscript.

### Conflict of interest statement

The authors declare that the research was conducted in the absence of any commercial or financial relationships that could be construed as a potential conflict of interest.
